# Stereometric and Tribometric Studies of Polymeric Pin and Ceramic Plate Friction Pair Components

**DOI:** 10.3390/ma14040839

**Published:** 2021-02-09

**Authors:** Magdalena Niemczewska-Wójcik, Artur Wójcik

**Affiliations:** 1 Faculty of Mechanical Engineering, Cracow University of Technology, Jana Pawła II 37, 32-864 Cracow, Poland; 2 Faculty of Production and Power Engineering, University of Agriculture in Cracow, Balicka 120, 30-149 Cracow, Poland; artur.wojcik@urk.edu.pl

**Keywords:** ceramics, polymers, surface topography, surface tribology, friction coefficient, wear intensity, wear products

## Abstract

Two complementary approaches should be used for the full characterisation of friction pair components. The first approach consists of stereometric studies of machined as well as worn surface topography of the friction components with multiple measurement methods used. The second approach, tribometric studies, enables the tribological characteristics of the friction pair. This work presents the complete characterisation of polymeric pin and ceramic plate friction pair components based on studies with the use of three research instruments: an interference microscope, a scanning electron microscope and a tribological tester. The results of the studies showed that the same treatment conditions used for different but similar ceramic materials did not provide exactly the same characteristics of both the machined and worn surface topography. Moreover, the results showed that the material properties and machined surface topography of the ceramic component significantly affected the friction coefficient and linear wear as well as the wear intensity of the polymeric component. Connecting the two approaches, stereometric studies and tribometric studies, allowed for a better identification of the wear mechanism of the polymeric pin (i.e., abrasion, fatigue and adhesion wear) and the kind of wear products (polymeric material).

## 1. Introduction

The properties of a surface layer shaped in the technological process play an important role in the operation process and determine the durability of the tribological system. The parameters of the technological process are selected in accordance with the materials and their properties in order to ensure the appropriate surface topography characteristics of the manufactured part in accordance with its intended use. The verification of the surface topography characteristic obtained in the technological process takes place during the operation process. Conducting observations of the operation process (including the identification of wear mechanisms and wear products) is limited due to the lack of access to the friction zone. For this reason, improving the tribological characteristics (including the friction coefficient, linear wear and wear intensity) take place through laboratory friction and wear studies (tribological tests) where it is possible to control the operation process at each stage.

The surface characterisation of the friction pair components should include two parallel approaches: surface topography measurements (stereometric studies) and tribological tests (tribometric studies) [1].

Stereometric studies allow for the determining of the functional properties of machined surfaces (for parts after the technological process) and worn surfaces (for parts after the operation process). These studies include the determination of parameters and functions characterising the surface topography [2,3]. They are carried out with the use of various measuring instruments and contact and non-contact methods (from macro to nanoscale) and their results enable multi-scale analysis [4,5,6].

The measurement instruments used for the study of surface topography allowing for its characterisation include, for example, a coordinate measuring machine (CMM) (shape and shape errors with quantitative and qualitative analysis), a scanning electron microscope (SEM) (surface morphology with qualitative analysis), an interference microscope (IM) (surface topography with quantitative and qualitative analysis) and an atomic force microscope (AFM) (surface topography and qualitative analysis) [7].

The surface topography characterisation presented in the works [8,9] were obtained by using multiple measurement instruments. Obtaining the completed information of the surface topography is possible by quantitative and qualitative description [2,3,7]. These descriptions allow the assessment of the machined surface and its properties [10,11,12,13,14,15] as well as an assessment of the worn surface (wear mechanisms and wear particles) of the friction pair components [16,17,18,19,20]. Moreover, the surface topography characteristics can be used to explain the wear mechanism of the friction pair components, for example in the Greenwood–Williamson model [21,22].

Tribometric studies have established the tribological properties of materials and friction pairs (tribological characteristics). There are various types of friction and wear studies, which include model tests (simplified cooperation conditions, elements of a friction pair with simple shapes) and simulation studies (mapping the real conditions of cooperation and the construction of the friction pair elements corresponding to real objects) [1,23]. These studies included recording changes in the friction coefficient and linear wear. The mean values of the friction coefficient as well as the wear intensity of the less durable component of the friction pair were then determined. The research presented in [24,25,26,27] shows that the results allow only the determining of the tribological characteristics such as the course of the variability of the friction force, the friction coefficient, the friction torque or wear intensity (including the determination of linear, volumetric or mass wear).

As such, the combination of these two approaches makes it possible to rationally solve research problems in the field of characterising the surface topography of friction pair components. Thanks to this, it is possible to optimise the functionality of the tribological system.

Various materials have been used as the friction pair components in the tribological system [23,28]. One example is ceramic materials, which are widely used in different tribological systems. Due to their properties, they are used, among others, in medicine (friction pair components of implants) [23,29,30,31]. Ceramic is a difficult to machine material but ceramic parts can be manufactured in conventional and unconventional machining processes [32,33,34,35]. The most frequently used method of machining, ensuring high efficiency and the accuracy of the manufacture of a ceramic object, is abrasive machining [36,37].

In the tribological system the parts made of ceramic materials often co-act with the polymeric parts (for example, in medicine this pair of materials is used on the friction components of the artificial hip joint). Polymeric material is a soft material that gradually wears out during the operation process. Hence, a significant influence on the durability of the tribological system in which the friction pair consists of a polymeric part and a ceramic part is that of the ceramic part and its surface topography characteristics [1,38].

While reviewing the literature, few publications about the comprehensive characterisation of friction pair components (including the authors’ own works [7,9,39]) were found concerning the studies of machined and worn surface topography as well as their assessment based on parametric and non-parametric descriptions.

Therefore, the present work focuses on the comprehensive characterisation of the friction pair composed of ceramic and polymeric components. The characterisation is based on the results of stereometric and tribometric studies that contribute to the primary expertise of this matter.

## 2. Materials and Methods

The studies and analysis are concerned with ceramic materials (monocrystalline (MON) and polycrystalline (POL) ceramics) co-acting with a polymeric material (ultra-high-molecular-weight polyethylene (UHMWPE)) in a friction pair consisting of a polymeric pin and a ceramic plate. These materials have obtained positive results in biomedical research (confirmed by certificates), thanks to which they can be used in medicine, for example on the friction components of an artificial hip joint. The characteristics of the studied materials are presented in Table 1.

The finishing process of the ceramic parts (for both MON and POL), an abrasive machining process, included two operations. The first operation, bonded abrasive machining (grinding), enabled making the shape and the third dimension of height. The second operation, loose abrasive machining (lapping), enabled making the quality of the surface topography and contained three treatments. The tool in these three treatments was diamond micropowder with a granulation ranging from 40 to 3 µm. In this work, the results of the last processing (precision lapping) in which the tool was diamond micropowder with a granulation ranging from 5 to 3 µm were presented. The ceramic parts (plates) with dimensions of 37 × 17 × 7 mm^3^ were characterised by two types of surface topography, B (for the treatment time of 5 min) and A (for the treatment time of 10 min).

The finishing process of the polymeric parts also included two operations, turning and polishing. The polymeric parts with a diameter *ϕ* 9 mm were characterised by one type of the surface topography of a working area. The details of the machining process for both friction parts are described in the work [7].

Two measurement instruments were used for the stereometric studies of the machined surface and worn surface topography, a white light interference microscope (WLIM) (Talysurf CCI Lite, Taylor Hobson Company, Leicester, UK) and a scanning electron microscope (SEM) (Quanta 3D FEG SEM, FEI Company, Hillsboro, OR, USA). Stereometric studies were performed on ten areas of each ceramic plate and five areas of each polymeric pin. Due to the repeatability and the number of obtained results, selected and representative results are presented in figures and tables. The surface topography obtained using WLIM was prepared for analysis by removing the measurement noise (spatial filtering (median denoising filter) for removing noise and spikes; filter size 3 × 3) and suitable filtering for the elimination of incorrect pits and/or peaks (threshold 1% from the top and 99% from the bottom, which ensured obtaining a statistically relevant effect). Moreover, the non-measurement points were filled with a smooth shape according to the neighbourhood.

Qualitative and quantitative descriptions of the surface topography of the friction pair components were performed. The quantitative (parametric) description was performed on the basis of 3D surface texture parameters (Sq, root mean square; Sp, maximum peak height; Sv, maximum valley depth; Ssk, skewness; Sku, kurtosis) by ISO 25178 [40]. The analysis of the surface texture parameters was possible by the usage of TalyMap Platinum v.7 software. The qualitative (non-parametric) description was carried out on the basis of the surface topography view (WLIM) as well as the surface morphology (SEM).

The tribological tests were performed with the use of a tribometric instrument (tribotester T-17, Łukasiewicz Research Network—The Institute for Sustainable Technologies, Poland) in Ringer’s solution. The tribological characteristics as a friction coefficient and wear intensity of a polymeric component for four kinds of friction pairs (pin-on-plate) were obtained:

1. Polymeric pin and monocrystalline ceramic plate type B: UHMWPE-MON(B);
2. Polymeric pin and monocrystalline ceramic plate type A: UHMWPE-MON(A);
3. Polymeric pin and polycrystalline ceramic plate type B: UHMWPE-POL(B);
4. Polymeric pin and monocrystalline ceramic plate type A: UHMWPE-POL(A).

Each tribological test was repeated three times using the new friction pair components (polymeric pins and ceramic plates). Due to the repeatability of the studies and the amount of data, this work presents the representative results.

The measurement methods with characteristics are presented in Table 2.

One of the elements of the control-measuring system in the tribotester T-17 is the lubricant heating and filtering system, which contains a filter made of a polymeric material. The Ringer’s solution from the friction zone flows through this filter. All pollution, including the wear particles, are retained on the filter surface. Hence, after tribometric studies, additional studies were conducted and the wear particles on the surface of the filters were identified using SEM.

Connecting both the stereometric and tribometric studies made it possible to provide the complete description of the polymeric pin and ceramic plate friction pair components including surface topography characteristics (machined and worn) and tribological characteristics (friction coefficient, linear wear, wear intensity and wear particles).

## 3. Results and Discussion

**Figure 1:**
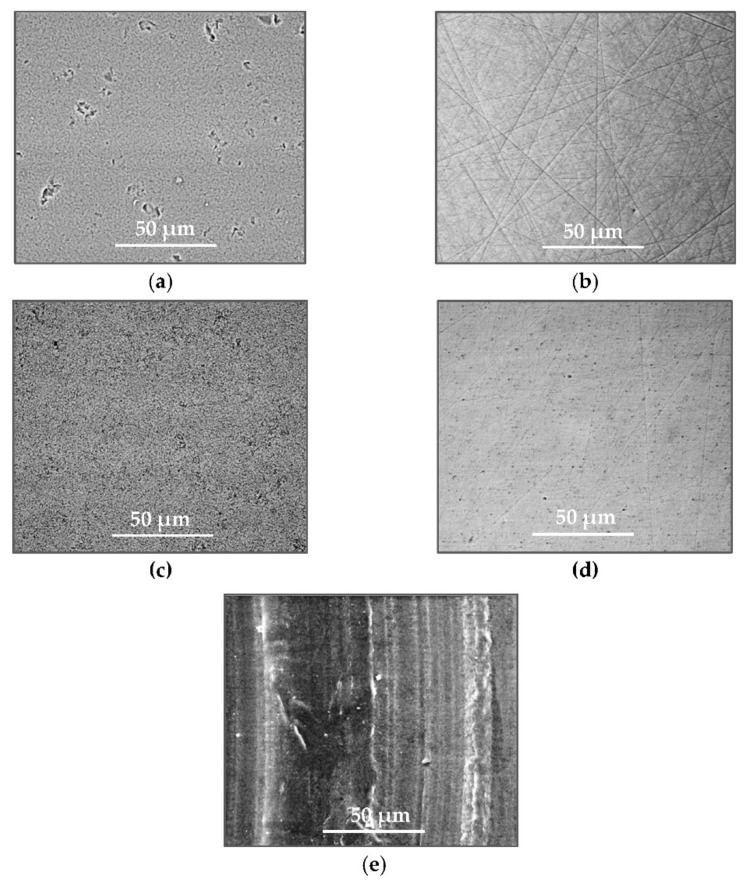
Stereometric studies of the machined surface (surface morphology) by SEM: (**a**) plate MON(B); (**b**) plate MON(A); (**c**) plate POL(B); (**d**) plate POL(A); (**e**) pin UHMWPE.

A surface analysis and characteristics of the friction pair components obtained from the finishing process (machined surface topography) were performed on the basis of the results presented in Table 3 (WLIM results; surface topography view and surface topography extraction view, surface texture parameters) and Figure 1 (SEM results; surface morphology).

Different types of material discontinuities (surface defects [7,43]) were observed on the surfaces of the ceramic plates.

The values of the Sq parameter for the MON(B) surface and the POL(B) surface as well as the MON(A) surface and the POL(A) surface were comparable. The surface texture parameters could be similar but differences were found in the type of local surface defects (scratches, crumbles, dents or pores).

In the case of the MON(B) surface, these consisted of numerous small valleys or dales (dents and crumbles). There were fewer dales on the MON(A) surface but their sizes were larger (depth up to 0.35 µm). These differences were presented in the surface topography view as well as in the surface topography extraction view; the value of the Sv parameter indicated the depth of the dales.

Apart from the dales on the MON surfaces, numerous scratches were also observed, which were the result of the interference of the tool (diamond micrograins) in the structure of the ceramic material during the machining process. The Sku parameter confirmed the presence of discontinuities on the MON surfaces of the studied plates. The higher value of this parameter indicated the occurrence of various discontinuities (surface defects; dales, scratches) on the surfaces and their chaotic distribution. The Ssk parameter characterised the type of surface shaping. Negative values indicated the plateau character of this shaping; extensive peaks as well as dales with gentle slopes.

Compared with MON(B), the Ssk parameter for MON(A) was almost two times smaller and the Sku parameter almost three times higher, which indicated that this surface was characterised by numerous unevenly distributed hills. On the POL surfaces, apart from scratches, numerous dales mostly with regular contours were also observed. In the case of the polycrystalline ceramics, these were pores (open and closed), which were characteristic of the sintered materials. The porous nature of polycrystalline ceramics limited the possibility of eliminating these types of anomalies in the treatment process.

The POL(A) surfaces were characterised by a smaller size of dales (lower value of the Sv parameter). As in the case of MON surfaces, the value of the Ssk parameter for POL surfaces was negative. However, the Ssk parameter for the POL surfaces was more than three times higher compared with the MON surfaces. On the other hand, the value of the Sku parameter was significantly lower compared with the MON surfaces, which indicated a lesser number of discontinuities. These differences were visible in the surface topography view as well as in the surface topography extraction view.

The presented surface shaping (surface topography characteristic) of the ceramic plates plays an important role in the operation process, determining the wear mechanism and wear intensity of the polymeric pin [7,44].

On the UHMWPE surface, clear traces of machining (evenly spaced circles) were visible and the depth of which did not exceed 4 µm. A positive value of the Ssk parameter indicated the presence of peaks and dales with steep slopes. Conversely, the value of the Sku parameter confirmed the character of the surface shaping presented in the surface topography view as well as in the surface topography extraction view.

The results obtained from the SEM showed that the MON surfaces were characterised by numerous dales (dents and crumbles; Figure 1a) and scratches (Figure 1b). On the POL surface, there were mainly small dales (pores; Figure 1c) and scratches (Figure 1d). On the UHMWPE surface (Figure 1e), evenly spaced circles of dales/peaks of different depths/heights were visible.

Additionally, the filters used in tribological tester (tribotester T-17) were studied. These studies were performed to determine the chemical composition of an unused filter (C, O, N) and to compare it with the results obtained after tribological studies.

After carrying out stereometric studies of the machined surface topography of the ceramic and polymeric parts and an unused filter, tribometric studies (friction and wear studies) were carried out.

The results (tribological characteristics) of the friction coefficient and the linear wear of the polymeric pin are presented in Table 4. Additionally, the mean value of the friction coefficient µ for each studied friction pair was determined.

During each tribological test, the polymeric pin was worn away (its value was presented as linear wear). Part of the polymeric material was transferred onto the ceramic plate (similar results for other friction pairs were presented in the works [27,39]). Therefore, assuming the wear of the ceramic plate was zero, the wear intensity I, of the polymeric pin was determined.

The analysis of the friction coefficient and linear wear showed differences not only resulting from the used materials but also from the surface characteristics of the ceramic plates (two types of surface topography A and B for MON and POL ceramic plates).

For the friction pairs UHMWPE-MON after the time of mutual running-in (200,000 cycles), a more stable run was recorded for the friction pair UHMWPE-MON(B). The friction coefficient in this case varied from 0.085 to 0.04; an average value was 0.059. For the friction pair UHMWPE-MON(A), the run was unstable and the friction coefficient varied from 0.02 to 0.09; an average value was 0.071. For the friction pairs UHMWPE-POL, in both cases, POL(A) and POL(B), after the time of mutual running-in, the average value of the friction coefficient was 0.08 for the friction pair UHMWPE-POL(B) and 0.07 for the friction pair UHMWPE-POL(A).

The average wear intensity was determined by linear wear (Table 4). The lowest wear intensity was recorded for the friction pair UHMWPE-MON(B) while the highest wear intensity was for the friction pair UHMWPE-POL(B).

After the tribometric studies, stereometric studies of the worn surfaces of the components of the friction pairs were performed. The results are presented for the ceramic parts in Table 5 and for the polymeric parts in Table 6.

On the surfaces of the ceramic plates, the polymeric particles (the main wear product in all of the pin-on-plate tests) were observed. The surface texture parameters of the ceramic plates changed due to the transfer of the polymeric material. The amount and distribution of wear particles was dependent on the surface topography characteristics of the ceramic plates obtained in the technological process. Typical for all ceramic surfaces was the decrease the value of the Sv parameter by about two-fold. As a result of friction, the dales on the ceramic surfaces were filled with the wear products (polymeric particles). In the case of the MON surfaces, as a result of the wear process, the Sv and Sku parameters decreased both for MON(A) and MON(B). However, the Sp and Ssk parameters increased (a greater change was noted for the MON(A)). In the case of the POL surfaces, the Sp, Sv and Sku parameters decreased and the Ssk parameter increased (a greater change was noted for the POL(B)).

On the surfaces of the polymeric pin after the operational process, horizontal traces were observed. These traces suggested that the irregularities (i.e., dales, peaks and their number and size) of the surface topography of the ceramic plate during the tribological test caused scratching on the surface of the polymeric pin. The quantitative analysis of the surface texture parameters characterising the worn surfaces of the polymeric pin showed mostly a decrease in their value compared with the parameters characterising the machined surfaces, which resulted from the operational process (wear of the polymeric pin).

The greatest change in the roughness parameters (especially the Sq, Sp, Sv and Ssk) was recorded for the surface of the UHMWPE pin co-acting with the MON(B) plate, the surface of which was characterised by the largest 3D machined surface texture parameters. As a result of friction, the irregularities on the UHMWPE surface were removed by the peaks of the MON(B) surface. The wear products (polymer particles) were collected in the dales on the ceramic plate surface. Ringer’s solution located in the dales on the MON(B) surface softened the wear process of the polymeric pin, which was confirmed by the tribological characteristics (µ and I; Table 4).

**Figure 2:**
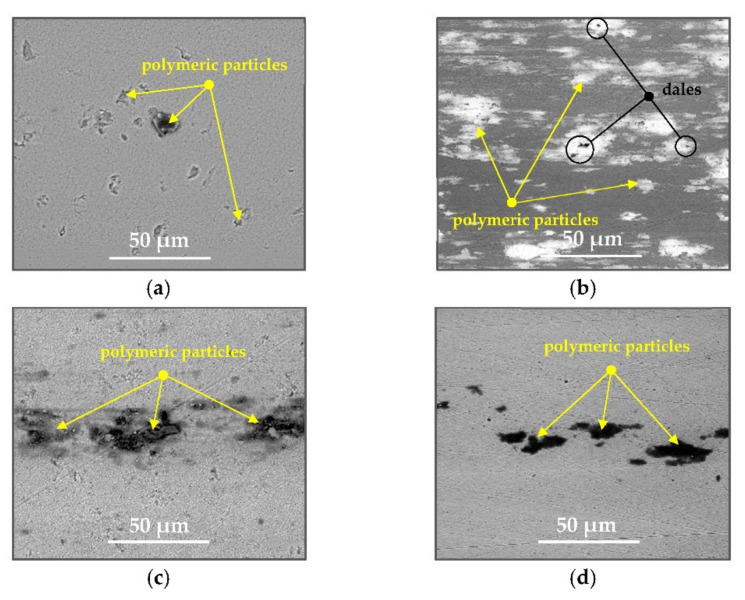
Stereometric studies of the worn ceramic surface (surface morphology, wear products) by SEM: (**a**) plate MON(B); (**b**) plate MON(A); (**c**) plate POL(B); (**d**) plate POL(A).

**Figure 3:**
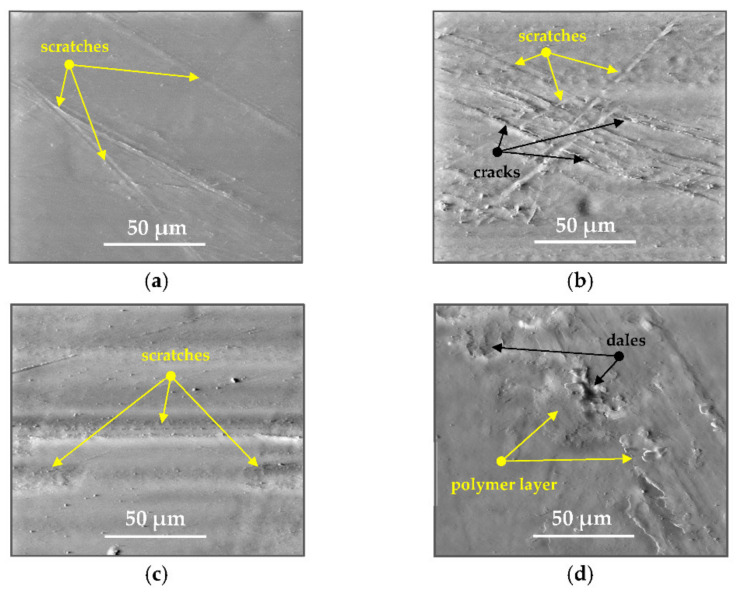
Stereometric studies of the worn polymeric surface (surface morphology, wear mechanism) by SEM: (**a**) UHMWPE_MON(B)_; (**b**) UHMWPE_MON(A)_; (**c**) UHMWPE_POL(B)_; (**d**) UHMWPE_POL(A)_.

The results obtained from the SEM reflected the real images of the worn surfaces, showing their morphology (Figure 2 (ceramic parts) and Figure 3 (polymeric parts)).

A qualitative analysis of the ceramic surfaces after the tribological tests showed that the largest amount of polymeric material (UHMWPE) was transferred to the MON(A) surface as bright traces. Small amounts of wear products (polymeric particles) were observed on the POL(A) surface; the darker the colour, the thicker the layer of the transferred polymeric material.

On the UHMWPE surface co-acting with the MON(B) surface, a few scratches were observed while more scratches were observed on the UHMWPE surface co-acting with the MON(A) surface. These scratches were produced as a result of microcutting by the irregularities of the MON surfaces (abrasion wear mechanism). In addition, other material discontinuities were observed, which were produced as a result of the long-time slide of the MON(A) surface on the UHMWPE surface (fatigue wear mechanism and cracks of polymer material). The wear mechanism of the UHMWPE surface co-acting with the POL surfaces was similar especially for the friction pair UHMWPE-POL(B). In the case of the friction pair UHMWPE-POL(A), the destruction of the polymeric UHMWPE surface occurred as a result of the adhesive interaction of the polymeric material transferred onto the ceramic surface with the polymeric material of the pin (adhesion wear mechanism). This was evidenced by the local dales on the UHMWPE surfaces. Moreover, other material discontinuities were also observed such as the delamination of the polymer material as a result of the fatigue wear mechanism (the layer of the polymer material on the surface of the polymeric pin).

**Figure 4:**
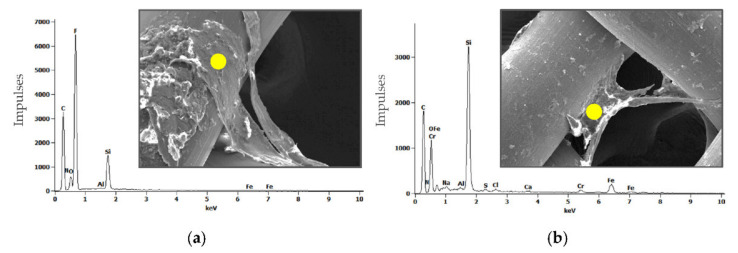
Images of filters after tribological tests (view of wear particles)—SEM with EDS spectrums: (**a**) friction pair UHMWPE-MON; (**b**) friction pair UHMWPE-POL.

Figure 4 shows the SEM results for the filters, which were performed after the tribological tests.

Qualitative (surface morphology, view of wear particles) and quantitative (chemical composition of wear products, EDS spectrums) analyses confirmed that the main wear product in the tribometric study of the polymeric pin and ceramic plate friction pairs regardless of the material and the surface topography characteristics of the ceramic plate was the polymeric pin material. Apart from the chemical elements of a clean, unused filter (C, O, N), the chemical elements of Ringer’s solution (Na, Cl, Ca) as well as small amounts of ceramic plate material (including Al, Cr, F, Fe, Si) were identified.

## 4. Conclusions

There was a certain correlation between the machined surface established in the technological process, tribological characteristics and the worn surface created during the operation process.

The use of the same treatment conditions for different but similar ceramic materials did not provide the same tribological characteristics of friction pairs, nor did it involve the same wear mechanisms of a polymeric component. This was best presented by the results obtained for the ceramic components (surfaces) of MON(A) and POL(A).

Connecting the stereometric and tribometric studies showed that the best polymer-ceramic friction pair in terms of the course of changes in the friction coefficient and linear wear of the polymeric component were the pairs UHMWPE-MON(B) and UHMWPE-POL(A).

The wear products (polymeric particles) visible on the surfaces of the ceramic components were the consequence of the wear mechanisms including abrasion and fatigue as well as adhesion wear.

Based on the presented results, simulation studies (friction pair parts corresponding to real objects [9] as well as tribometric studies in real conditions) are planned to be conducted.

## Figures and Tables

**Table 1:** The characteristics of the materials of friction pairs [1,7,28].

Properties	Ultra-High-Molecular-Weight Polyethylene (UHMWPE)	Monocrystalline (MON) Ceramic	Polycrystalline (POL) Ceramic
Density [g/cm^3^]	0.94	3.99	6.05
Young’s Module [GPa]	0.75	400	209
Poisson’s Ratio [–]	0.46	0.27 ÷ 0.30	0.23 ÷ 0.32
Chemical	C > 90%	Basis: Al_2_O_3_	Basis: ZrO_2_
composition wt.%	O < 10%	Fe_2_O_3_ < 0.05%	Y_2_O_3_ < 5.3%
		Cr_2_O_3_ < 0.03%	Al_2_O_3_ < 0.05%
		TiO_2_ < 0.01%	Fe_2_O_3_ < 0.01%
			SiO_2_ < 0.02%

**Table 2:** The characteristics of the measurement methods.

**Stereometric Studies**	
**White Light Interference Microscope WLIM**	**Scanning Electron Microscope** **SEM**
- lens: Mirau 10× - measuring area: 1.65 × 1.65 mm^2^ - analysis: qualitative (images) and quantitative (parameters)	- module: LV (Low Vacuum) - magnification: 2000× - analysis: qualitative (SEM) and quantitative (EDS spectrum)
**Tribometric Studies** (ASTM F732-00 [41])—Tribotester T-17 (pin-on-plate)	
- motion type: sliding and reciprocating - contact geometry: plane - stroke length: 12.5 mm - load: 225 N - number of cycles: 10^6^ (about 12 days) - Ringer’s solution temperature: 36.7 ± 0.5 °C - Ringer’s solution: chemical composition: NaCl, CaCl_2_, KCl, NaHCO_3_ pH = 7.0 viscosity 12.2 cp [42]	

**Table 3:** Stereometric studies of the machined surface by WLIM.

Parameters	Surface Topography View	Extraction of Surface Topography View
**MON(B)** Sq = 0.116 µm Sp = 0.161 µm Sv = 0.904 µm Ssk = −4.39 Sku = 27.20	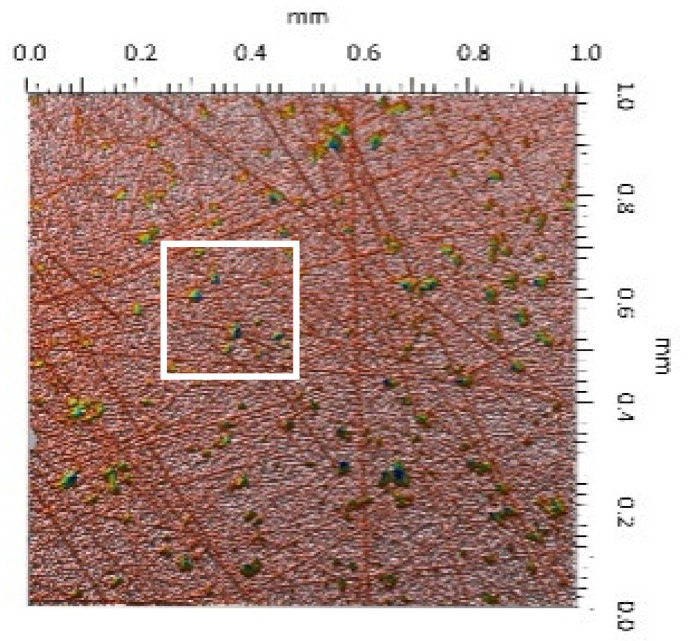	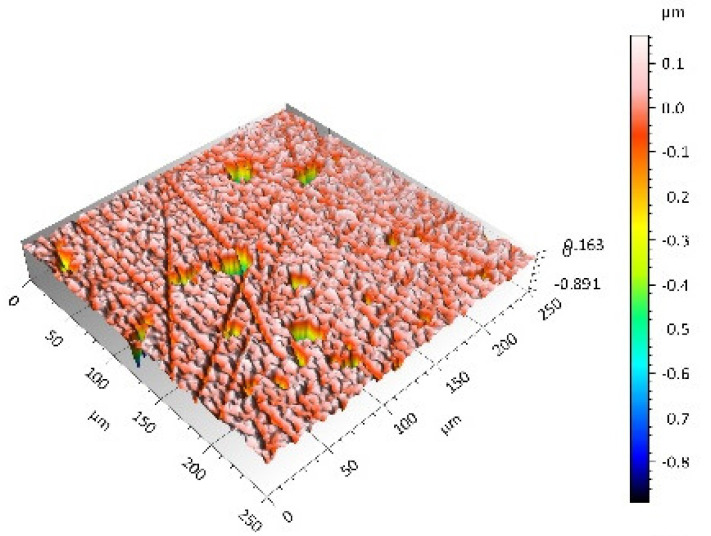
**MON(A)** Sq = 0.031 µm Sp = 0.049 µm Sv = 0.350 µm Ssk = −7.71 Sku = 70.30	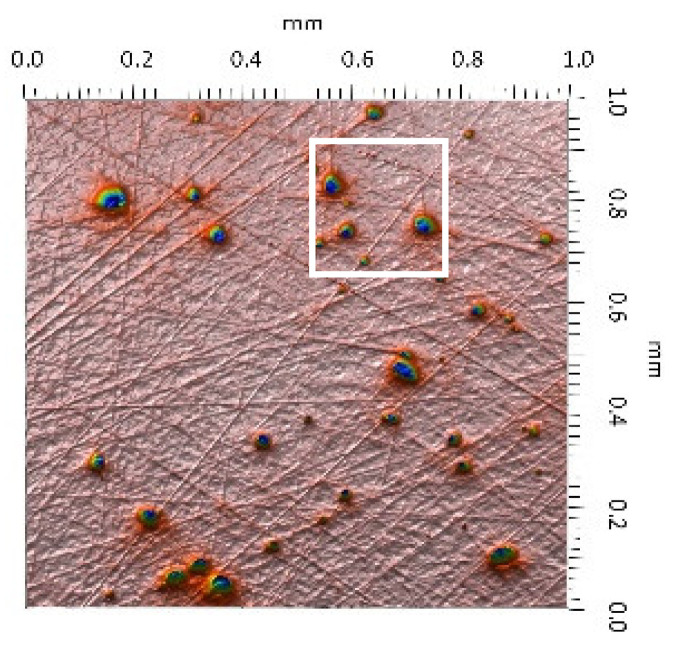	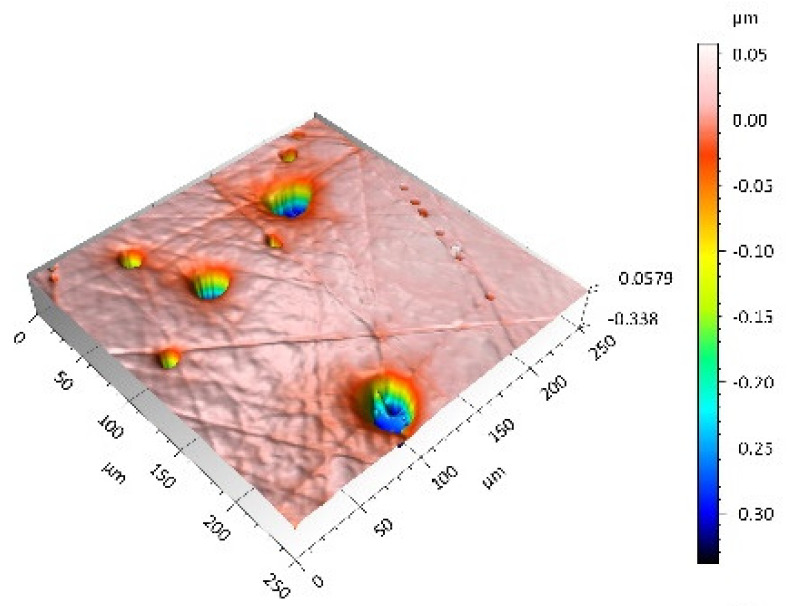
**POL(B)** Sq = 0.093 µm Sp = 0.244 µm Sv = 0.637 µm Ssk = −1.95 Sku = 8.80	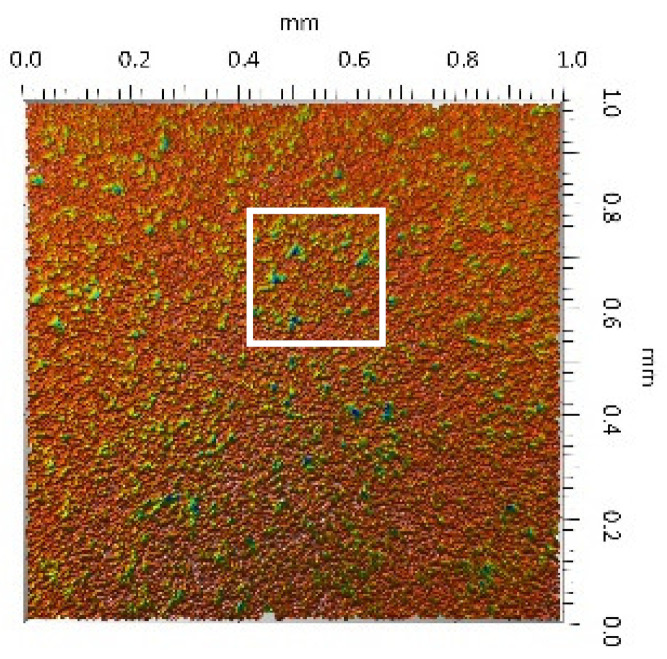	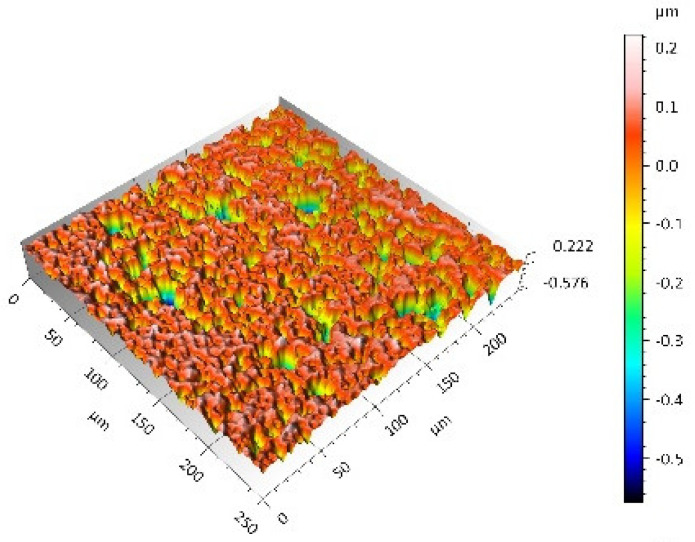
**POL(A)** Sq = 0.036 µm Sp = 0.146 µm Sv = 0.251 µm Ssk = −2.12 Sku = 14.00	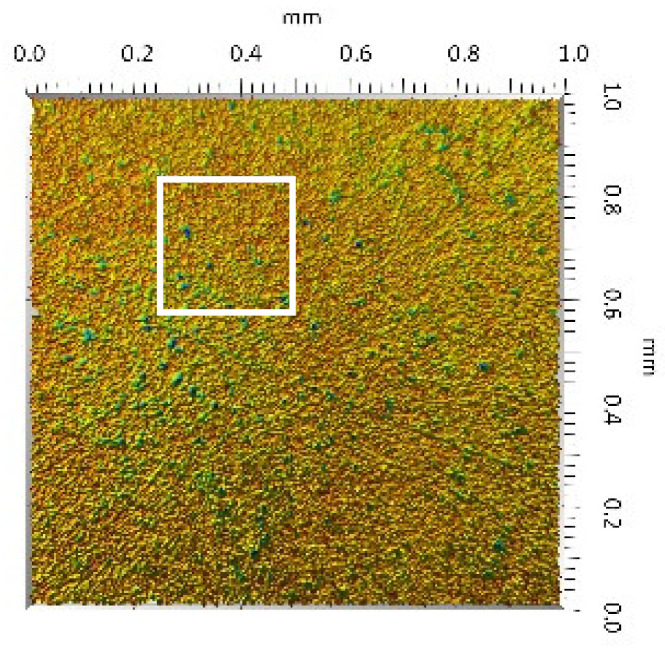	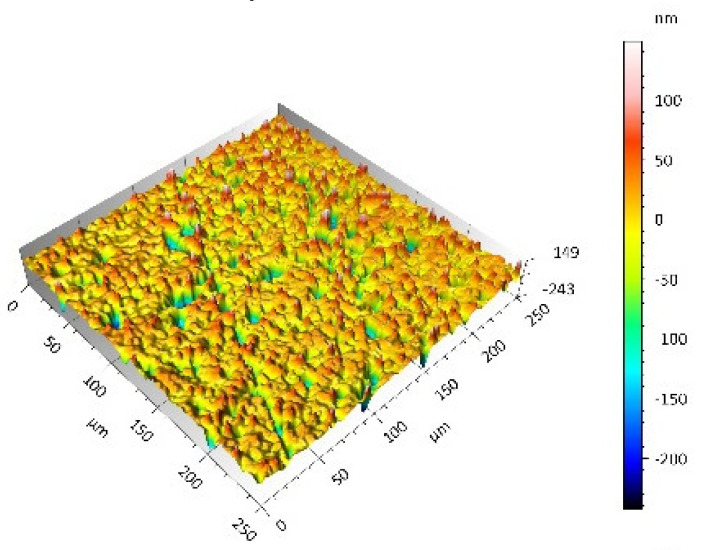
**UHMWPE** Sq = 1.690 µm Sp = 4.880 µm Sv = 2.870 µm Ssk = 0.56 Sku = 2.16	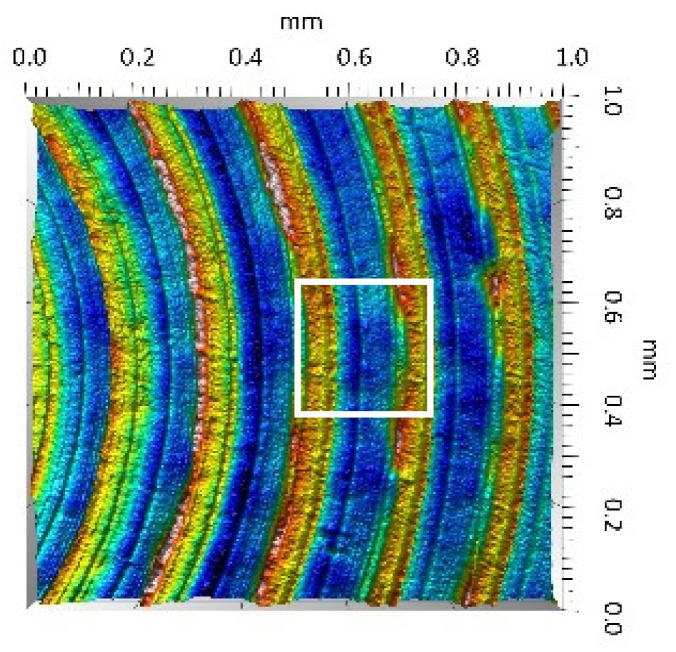	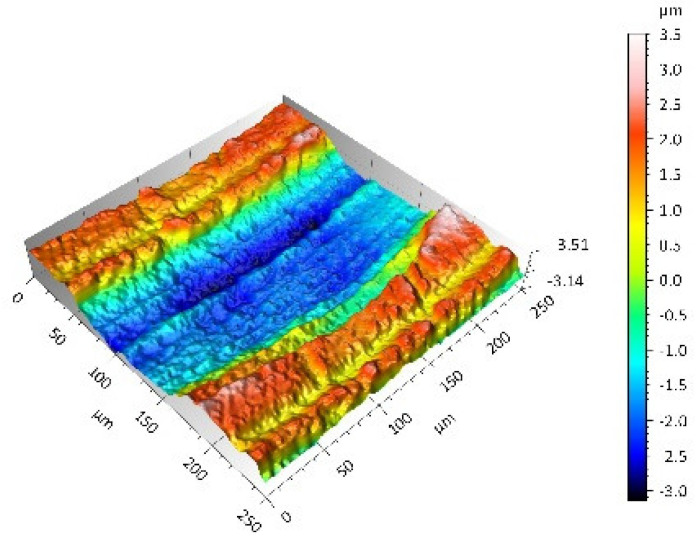

**Table 4:** Tribometric studies: the friction coefficient and linear wear of the polymeric components.

UHMWPE-MON	UHMWPE-POL
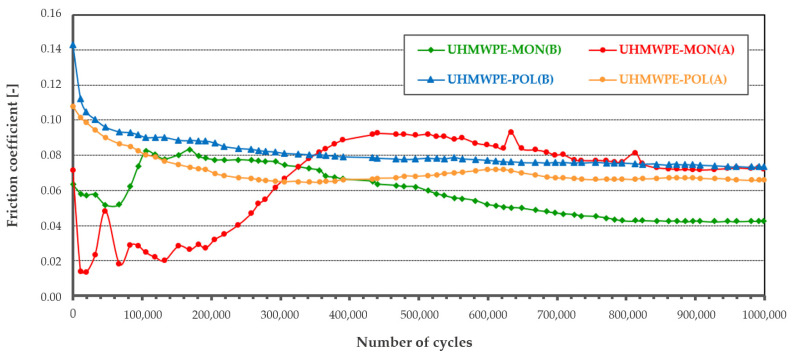	
**µ**_MON(B)_ = 0.059 ± 0.0135	**µ**_POL(B)_ = 0.076 ± 0.0173
**µ**_MON(A)_ = 0.071 ± 0.0245	**µ**_POL(A)_ = 0.067 ± 0.0124
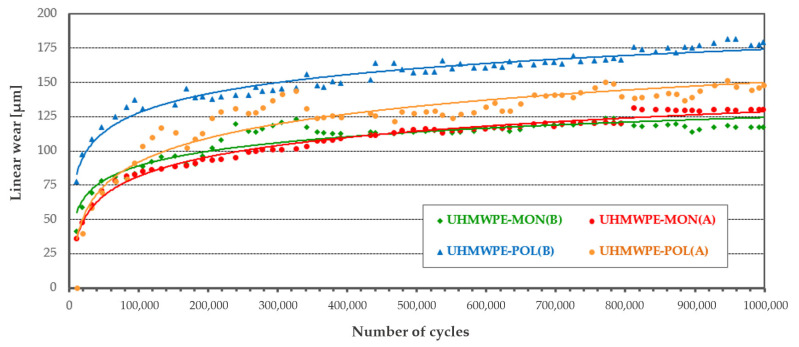	
**I**_MON(B)_ = 12.1 ± 0.3 µm/10^6^ cycles	**I**_POL(B)_ = 51.5 ± 7.0 µm/10^6^ cycles
**I**_MON(A)_ = 49.4 ± 3.0 µm/10^6^ cycles	**I**_POL(A)_ = 27.6 ± 4.0 µm/10^6^ cycles

**Table 5:** Stereometric studies of the worn ceramic surface by WLIM.

Parameters	Surface Topography View	Extraction of Surface Topography View
**MON(B)** Sq = 0.069µm Sp = 0.331 µm Sv = 0.511 µm Ssk = −3.20 Sku = 21.60	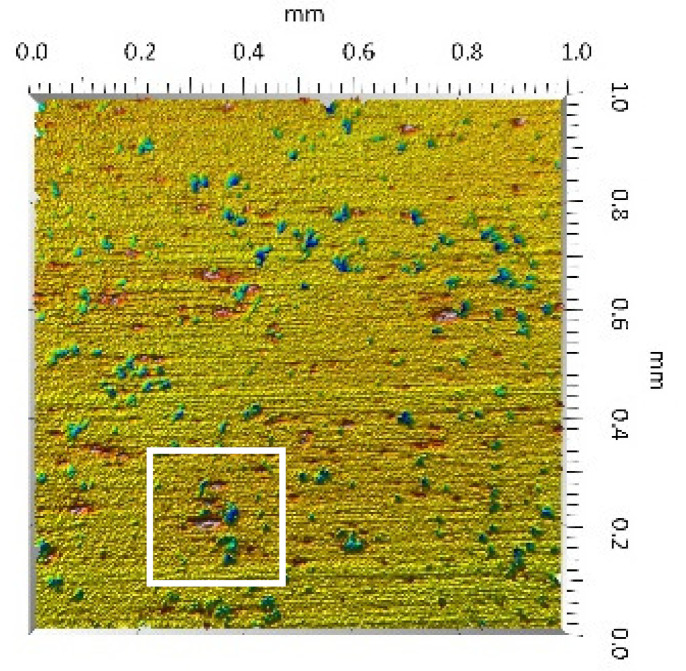	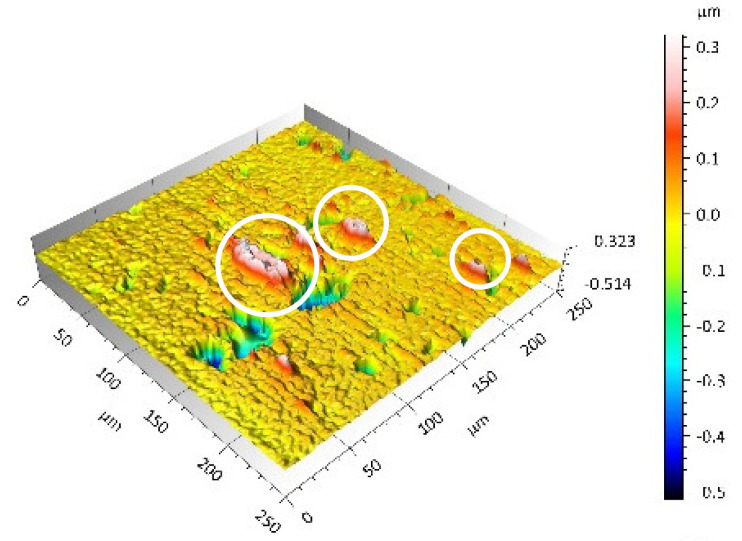
**MON(A)** Sq = 0.088µm Sp = 0.517 µm Sv = 0.199 µm Ssk = 2.66 Sku = 11.90	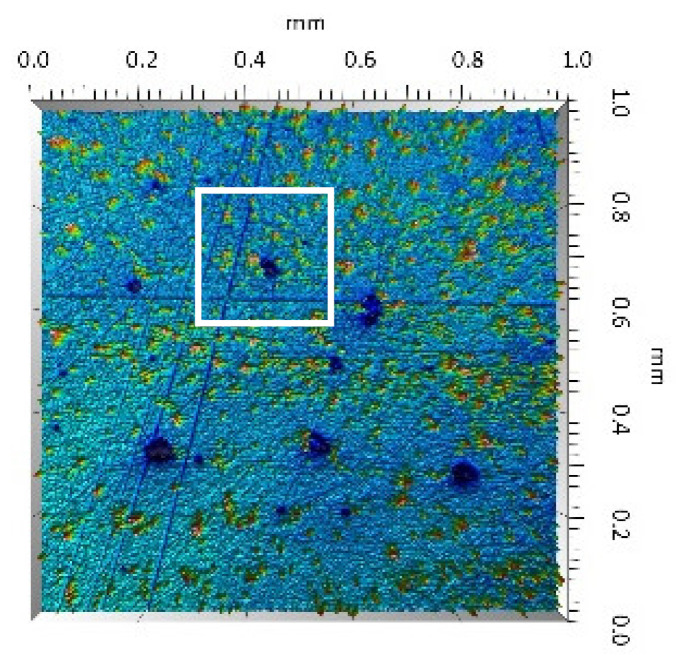	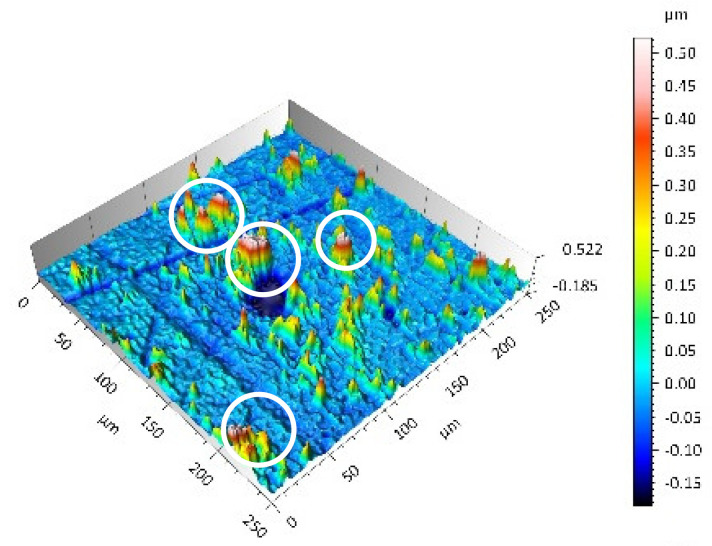
**POL(B)** Sq = 0.064 µm Sp = 0.196 µm Sv = 0.324 µm Ssk = −1.07 Sku = 4.70	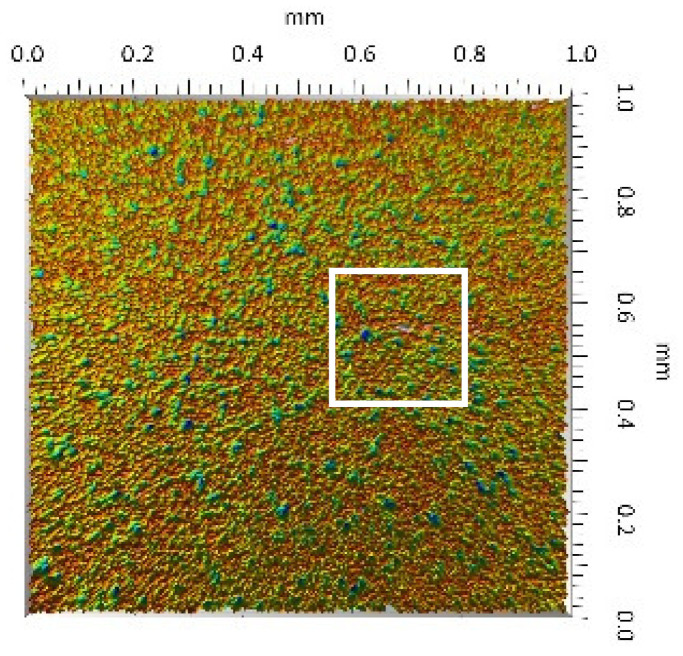	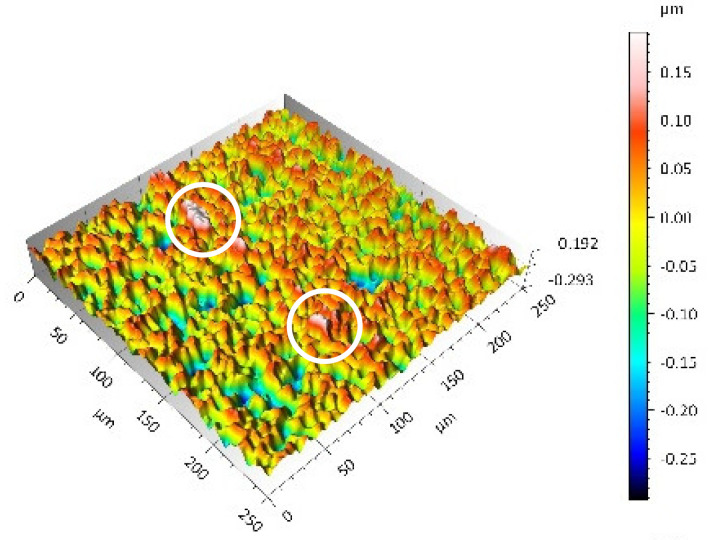
**POL(A)** Sq = 0.027 µm Sp = 0.095 µm Sv = 0.159 µm Ssk = −1.02 Sku = 5.39	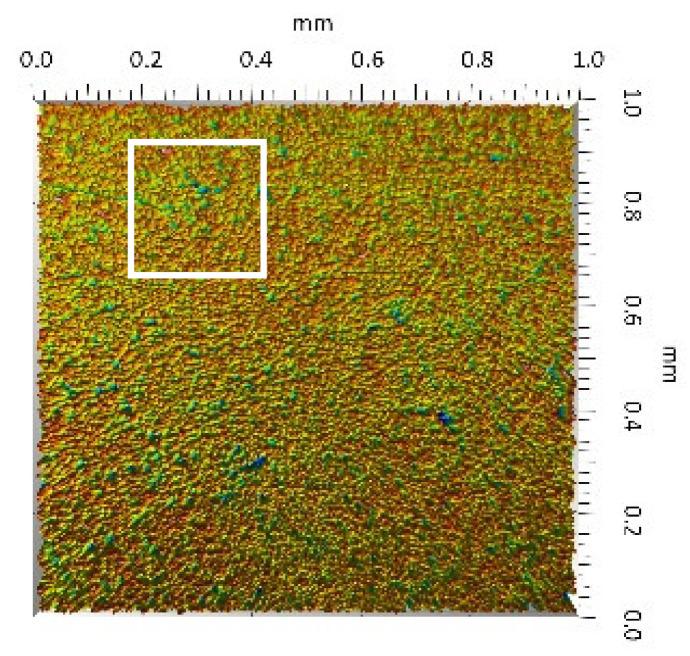	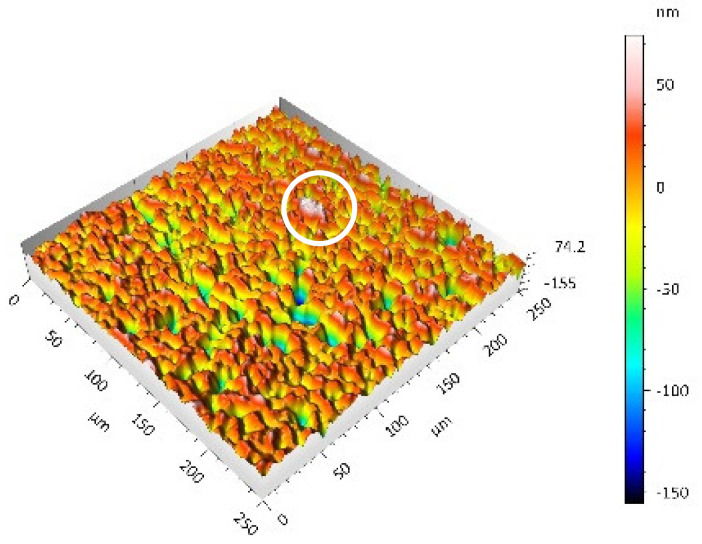

**Table 6:** Stereometric studies of the worn polymeric surface by WLIM.

Parameters	Surface Topography View	Extraction of Surface Topography View
**UHMWPE_MON(B)_** Sq= 0.407 µm Sp = 1.11 µm Sv = 1.20 µm Ssk = −0.11 Sku = 2.72	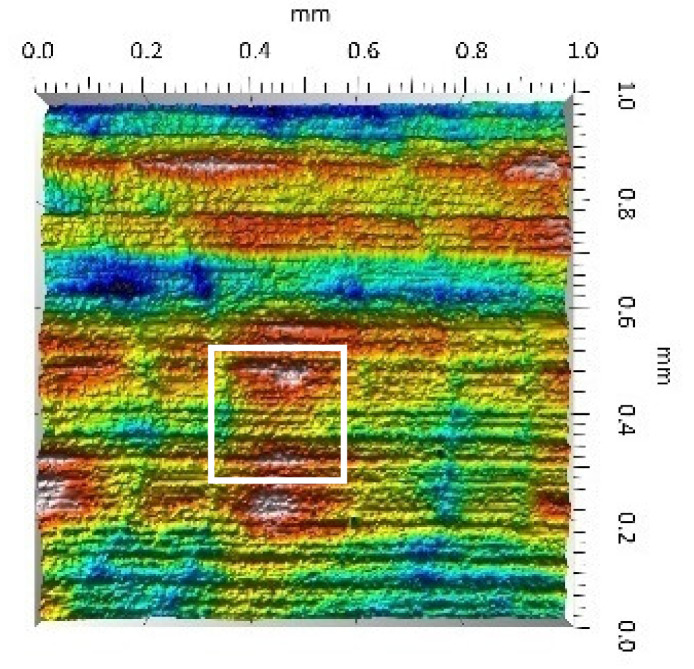	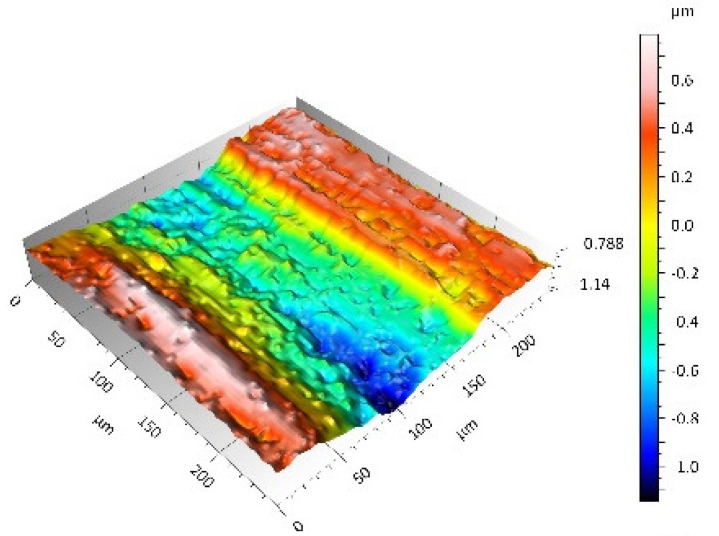
**UHMWPE_MON(A)_** Sq = 1.170 µm Sp = 2.73 µm Sv = 3.37 µm Ssk = −0.36 Sku = 2.62	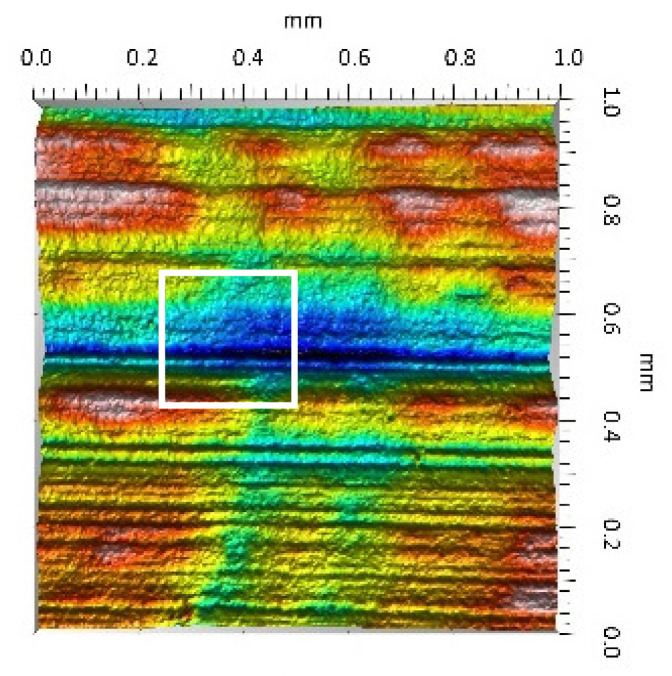	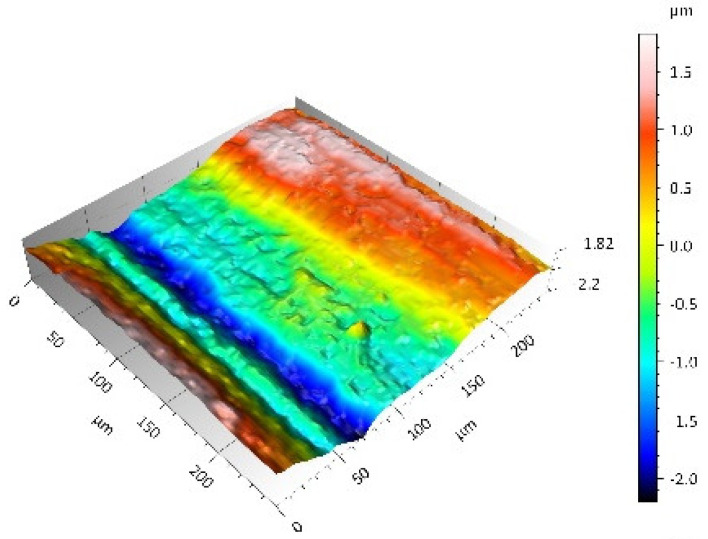
**UHMWPE_POL(B)_** Sq = 1.400 µm Sp = 5.13 µm Sv = 2.57 µm Ssk = 0.767 Sku = 3.12	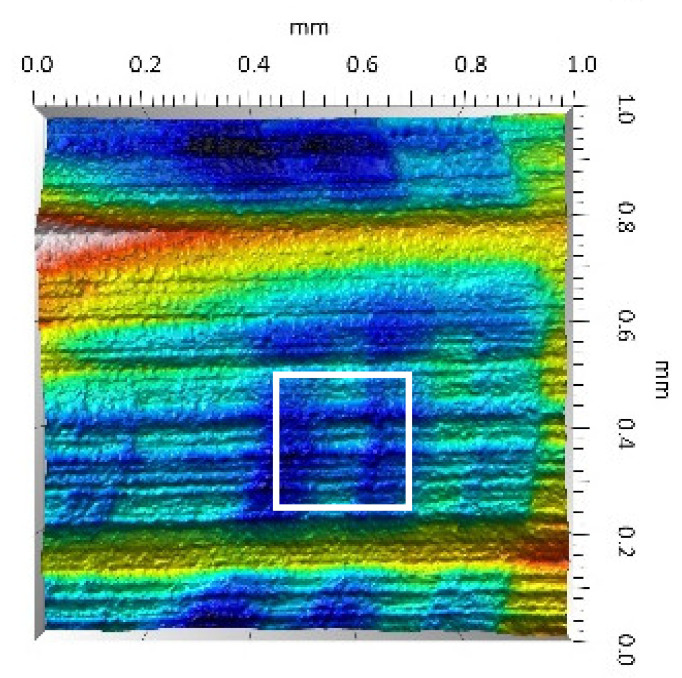	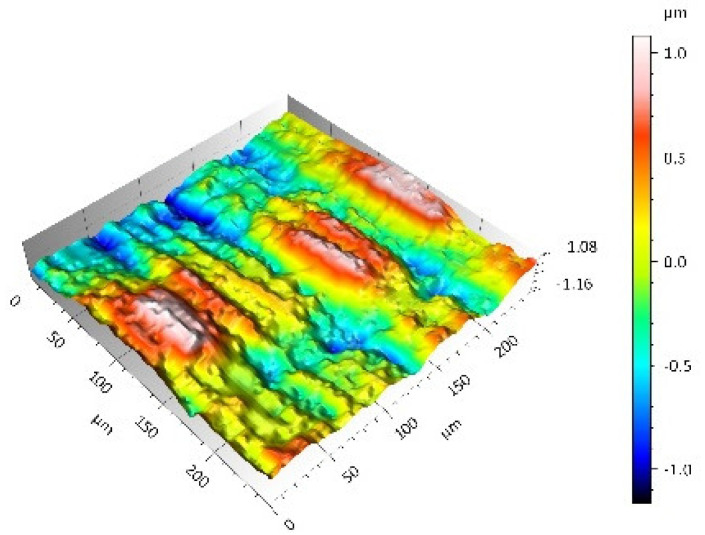
**UHMWPE_POL(A)_** Sq = 1.300 µm Sp = 4.19 µm Sv = 2.81µm Ssk = 0.488 Sku = 3.00	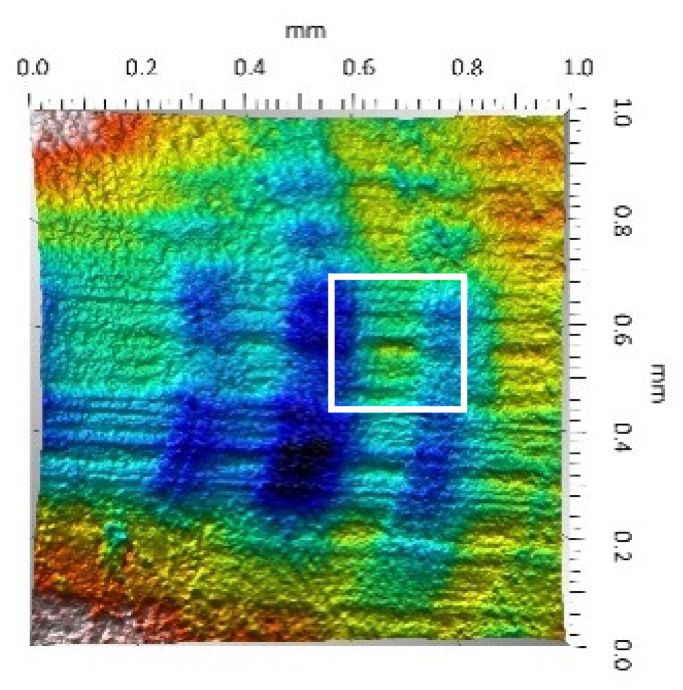	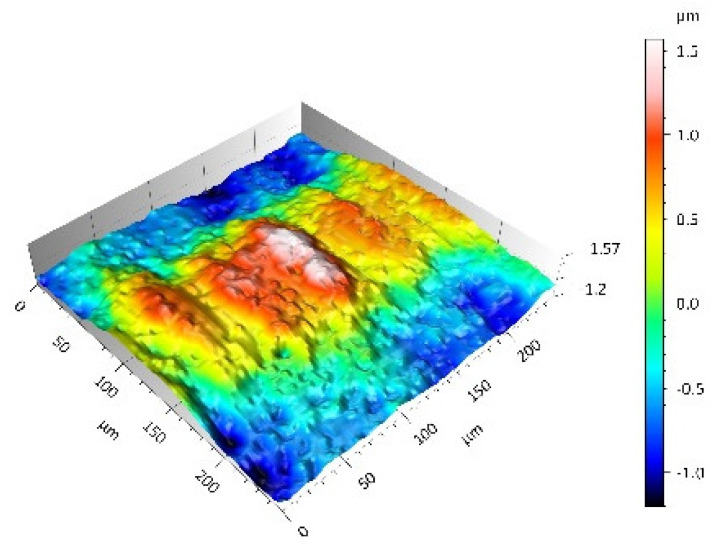

## Data Availability

Data is contained within the article.
